# Defining Variations in the Size of Normal Pituitary Glands and Pituitary Macrotumors Based on Canine Skull Morphology

**DOI:** 10.1111/vru.70135

**Published:** 2026-01-17

**Authors:** Hannah V. Pham, Kelsey D. Brust, Jessica A. Lawrence, Matthias Rosseel, Michael S. Kent

**Affiliations:** ^1^ Veterinary Medical Teaching Hospital University of California Davis School of Veterinary Medicine Davis California USA; ^2^ Department of Veterinary Surgery and Radiology University of California Davis School of Veterinary Medicine Davis California USA

**Keywords:** canine, CT, macroadenoma, macrotumor, pituitary

## Abstract

Computed tomography (CT) is a common imaging modality used to evaluate for a pituitary macrotumor in dogs. However, a standard definition of a pituitary macrotumor based on imaging characteristics has not yet been established. The human definition of a pituitary mass that measures >1 cm in diameter has previously been adopted for dogs but fails to account for variability in size, breed, and skull conformation. We hypothesized that normal pituitary gland size and macrotumor size vary by skull morphology. We also hypothesized that canine pituitary macrotumors may be smaller than 1 cm. In this retrospective, case–control analytical study, contrast CT scans from 89 dogs with imaging‐diagnosed pituitary macrotumors and 89 dogs with normal pituitary glands were compared. The height, length, width, and volume of the pituitary gland, sella turcica, and brain were measured along with the brain area at the level of the pituitary gland. Of the pituitary macrotumors, 21.3% (19/89) were smaller than 1 cm in height. Data support that a pituitary gland height >0.60 cm or a pituitary volume of 0.17 cm^3^ may represent a macrotumor in brachycephalic dogs, and a pituitary gland height >0.65 cm or a pituitary volume of 0.31 cm^3^ may represent a macrotumor in mesocephalic dogs; small numbers of dolichocephalic dogs precluded determination of cutoffs. These data provide a foundation for future studies to classify pituitary macrotumors on CT imaging according to skull conformation, thereby aiding in the detection, treatment, and response assessment of dogs with pituitary neoplasms.

**Abbreviations**
SI: skull index

## 1 Introduction

The pituitary gland is located ventral to the brain and sits in the sella turcica of the basisphenoid bone and plays a crucial role in maintaining homeostasis through its neuroendocrine functions. Pituitary tumors, which can be categorized as microtumors or macrotumors, are usually classified as adenomas but can be classified as carcinomas in rare situations where there is evidence of metastatic spread or osseous involvement [1]. In dogs, most pituitary macrotumors are derived from corticotroph cells. Pituitary macrotumors have been associated with endocrinological abnormalities due to disturbances in the production, storage, and release of hormones and neurological abnormalities due to direct compression of intracranial structures [1]. Contrast computed tomography (CT) is routinely used to objectively assess pituitary gland size, but precise definitions for pituitary macrotumors based on pituitary gland dimensions are lacking in veterinary medicine. It is critical to establish pituitary size cutoffs to improve early diagnosis, stratification of treatment options, and identify potential prognostic factors that may indicate more treatment‐resistant tumors.

A proposed definition translated from human literature characterizes pituitary macrotumors as a pituitary mass greater than 1 cm in diameter [2, 3]. A historical radiographic and surgical convention (Hardy–Wilson classification) separated intrasellar lesions from those sufficiently large to extend outside the sella; notably, this classification was done prior to standard magnetic resonance imaging (MRI) [4]. Nonetheless, the cutoff of 1 cm has been widely propagated into neurosurgical and endocrine guidelines. Contrary to humans, dogs have an incomplete diaphragmatic sella turcica and normal pituitary glands well under 1 cm, with a mean pituitary height reported as 3.8 mm on CT and 5.1 mm on 1.5 T MRI [5, 6, 7]. Another proposed definition characterizes a pituitary macrotumor as any pituitary mass that protrudes above the sella turcica [8, 9]. One of the primary challenges has been defining a macrotumor while accounting for the variability in dog size, breed, and skull conformation. One classification system that attempts to normalize pituitary size to skull morphology uses the ratio between pituitary height and brain area (P:B ratio) to account for differences in body weight [5, 8, 10, 11]. The P:B ratio correlated to endocrinopathy and presence of clinical signs, and a P:B ratio >0.31 mm^−1^ has been proposed as a cutoff for differentiating macrotumors from microtumors, although this has not been a consistent prognostic factor following treatment [8, 9, 12]. However, the P:B ratio has limitations, including that it requires additional time to calculate, misses a significant fraction of functional, dexamethasone‐resistant microadenomas, is a two‐dimensional height metric that may not capture extension within the cranial vault and may be influenced by skull conformation, brain atrophy, acute hemorrhage or edema, and potential image acquisition factors [11, 13, 14, 15, 16]. Additional work is therefore warranted to define metrics that may impact surgery and radiation therapy planning, such as rostro‐caudal extension.

By evaluating variations in the size of normal pituitary glands and pituitary macrotumors in dogs across different skull morphologies, a more precise definition of pituitary macrotumor dimensions on CT can be established for each skull morphology that may aid in the design and stratification of dogs in future studies. Although MRI is considered the gold standard for evaluating small pituitary lesions due to its superior soft tissue resolution, CT is often used in veterinary medicine for screening, as it has several advantages over MRI, including its speed, lower cost, and broad availability, as well as its use for radiotherapy planning purposes. We hypothesized that the size, defined as pituitary height and volume, of normal canine pituitary glands and pituitary macrotumors varies by breed and skull morphology: brachycephalic, dolichocephalic, and mesocephalic. Our secondary hypothesis was that pituitary height cutoffs may be stratified by skull morphology to better distinguish canine pituitary macrotumors from normal glands or microtumors.

## 2 Materials and Methods

### 2.1 Selection and Description of Subjects

This single‐center, retrospective, and case–control analytical study included pre‐ and post‐contrast CT scans of dogs from the electronic medical record database of the UC Davis William R. Pritchard Veterinary Medical Teaching Hospital between January 1, 2010 and March 31, 2024. Dogs diagnosed with pituitary macrotumor on contrast CT scans were included as pituitary cases. Dogs with pituitary tumors were included if they had pituitary masses measuring 1 cm or greater in height, or that had clinical signs attributed to a prominent pituitary gland that measured <1 cm in height, as per previously published studies. Controls were defined as dogs with normal appearing pituitary glands on contrast CT scans that did not have clinical signs consistent with hypercortisolism. Controls were breed‐matched to the cases, but if a perfect breed match was unavailable in the database, controls were matched by skull morphology. To find controls that met these criteria, the database was initially searched for contrast CT scans of dogs with nasal tumors, excluding stage IV tumors. The search was later expanded to include contrast CT scans of dogs with other tumors that did not affect the brain. Patients were excluded from the study if the CT scan did not capture the entire skull length (from prosthion to inion), and CT localizer images were not available for the skull length measurement. All case and control datasets were reviewed by an ACVR‐certified veterinary radiologist (K.D.B.).

Data use was approved by the institution, and approval by the Institutional Animal Care and Use Committee was not needed due to the retrospective design of this study. The search and clinical data extraction were performed by a third‐year veterinary student (H.V.P.) and approved by an ACVR board‐certified radiation oncologist (M.S.K.) and, following a second opinion review, blinded from the initial report, an ACVR‐certified veterinary radiologist (K.D.B.).

### 2.2 Data Recording and Analysis

CT image measurements were acquired by a third‐year veterinary student (H.V.P.) after training by an ACVR‐certified veterinary radiologist (K.D.B.). When the margins of structures were not readily identifiable, an ACVR‐certified veterinary radiologist (K.D.B.) was consulted to verify the measurements obtained or to determine if the case should be excluded due to inadequate quality for accurate interpretation. A commercially available radiation therapy treatment planning software (Eclipse, version 16, Varian Medical Systems, Palo Alto, CA, USA) was used for image evaluation and measurements.

**FIGURE 1:**
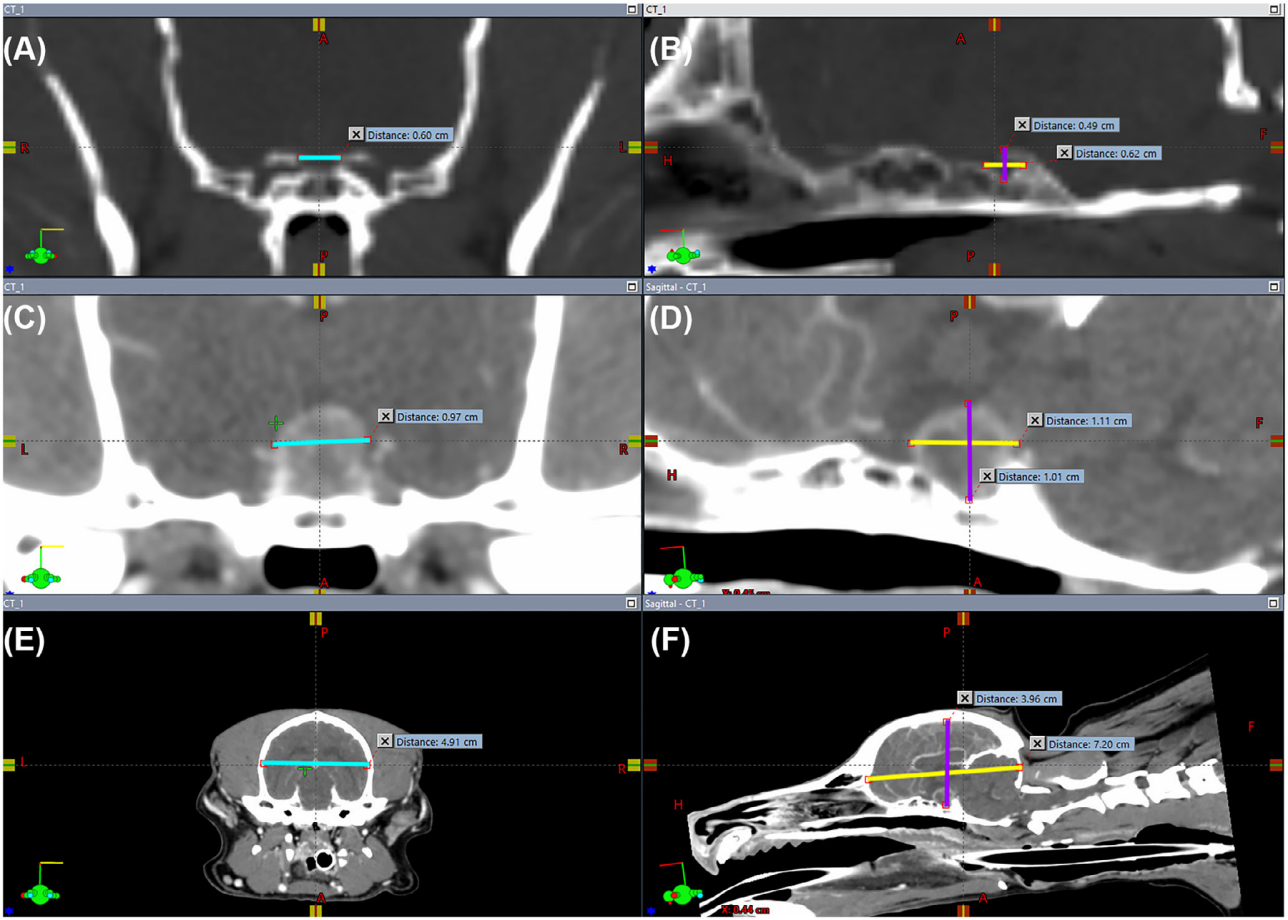
Sagittal (A, C, and E) and transverse (B, D, and F) CT images of a representative canine case from the study. (A) Sella turcica height (purple line) measured at the point of maximal height of the dorsum sellae, and sella turcica length (yellow line). (B) Sella turcica width (blue line). (C) Pituitary gland height (purple line) measured perpendicular to the basisphenoid bone, and pituitary gland length (yellow line). (D) Pituitary gland width (blue line). (E) Brain height (purple line), and brain length (yellow line). (F) Brain width (blue line). CT, computed tomography.

**FIGURE 2:**
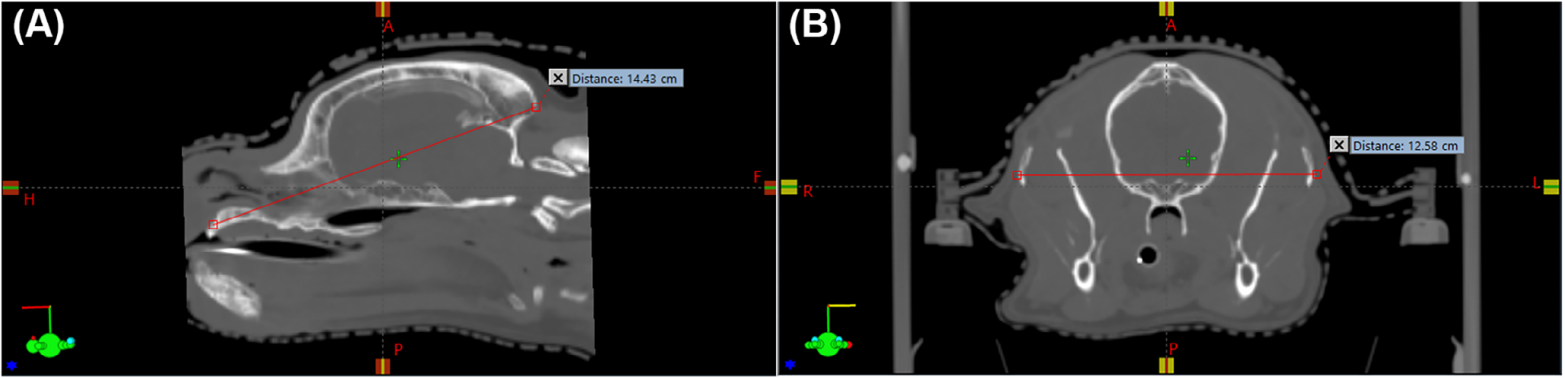
Representative images from a control dog, illustrating sagittal (A) and transverse (B) CT images of a canine skull. (A) Skull length measured from the prosthion (point a) to the inion (point b). (B) Skull width measured at the widest distance between the zygomatic arches.

Linear measurements of the skull and sella turcica were obtained on pre‐contrast CT scans displayed using a standardized bone window (window width [WW]: 100, window level [WL]: 200), and linear measurements of the pituitary gland and brain were obtained on post‐contrast CT scans displayed using a standardized soft tissue window (WW: 350, WL: 50). The observers were able to manually adjust the WW and WL if they deemed it necessary for more accurate interpretation. For the sella turcica, pituitary gland, and brain, the following linear measurements expressed in centimeters (cm) were obtained: maximal height and maximal length measured on the sagittal plane, and maximal width measured on the transverse plane (Figure 1). Landmarks for the pituitary gland were defined ventrally by the sella turcica border as well as dorsally, cranially, caudally, and laterally by the extent of the attenuation characteristics of the pituitary gland relative to the adjacent brain parenchyma, typically aided by the contrast enhancement within the gland. Landmarks for the sella turcica were defined by the depression in the basisphenoid bone, defined by the caudal clinoid processes, the hypophysial fossa, and the dorsum sellae. The landmarks for the brain were defined by the borders formed by the interior surface of the skull, which included the cerebrum, cerebellum, and brainstem rostral to the foramen magnum. For the skull, linear measurements expressed in cm included: skull length, defined as the distance from the prosthion to inion measured on the sagittal plane, and skull width, defined as the widest distance between the zygomatic arches measured on the transverse plane (Figure 2). A CT localizer image was used to evaluate the skull length for cases where the prosthion or inion was not captured in the CT scan. The skull index (SI) was calculated with the following equation: SI = (skull width (cm) × 100/skull length (cm)). Dogs were categorized by skull morphology according to SI based on the classification by Czeibert, where skulls with an SI < 51 were classified as dolichocephalic, 51 ≤ SI < 59 as mesocephalic, and 59 ≤ SI as brachycephalic [17].

**FIGURE 3:**
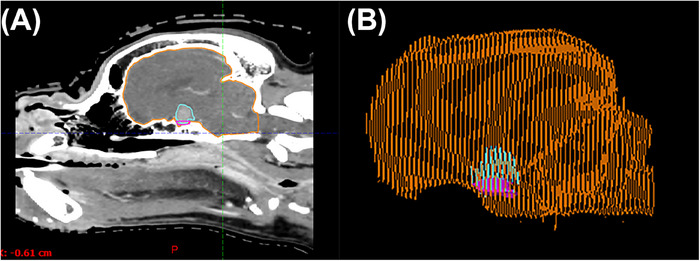
Representative images from a control dog, illustrating a sagittal contrast CT image (A) and volumetric rendering (B) of a canine skull with contoured structures: sella turcica (magenta), pituitary gland (blue), and brain (orange).

Volumetric measurements of the sella turcica, pituitary gland, and brain were obtained on post‐contrast CT scans displayed, using the standardized soft tissue window, although observers adjusted WW and WL if necessary. Each of the three structures was contoured, and volume was calculated by Eclipse (Figure 3). The area of the brain was measured via a hand‐drawn region of interest at the level of the pituitary gland, using Enterprise Imaging XERO Viewer (version 8.2.2.022 AGFA HealthCare Enterprise Imaging, Agfa HealthCare N.V. Septestraat 27 B‐2640 Mortsel, Belgium), to calculate the P:B ratio using the previously published Equation (6).

### 2.3 Statistics

The data were recorded in a commercially available electronic spreadsheet (Microsoft Excel 2024 for Mac, Version 16.90.2, Microsoft Corporation, Redmond, WA, USA). Statistical analyses were conducted by a third‐year veterinary student (K.D.B.) and an ACVR certified radiation oncologist (M.S.K.) using a commercially available statistics program (Stata version 14.2, Stata Corporation, College Station, TX, USA). Continuous data were checked for normality by a visualization of distributional plots and using the Shapiro–Wilk test. When normally distributed, continuous data were reported with means and standard deviations. Otherwise, medians with interquartile ranges and/or overall ranges were reported. Comparisons of dimensions, P:B ratio, and volumes between groups were evaluated using ANOVA with Bonferroni correction for multiple‐comparison tests or Kruskal–Wallis tests, depending on data normality, to look for differences between groups. Receiver operator characteristics (ROC) curve analyses were performed for pituitary height and volume for each skull morphology. Optimal threshold values were selected via the Liu method, which maximizes the product of the sensitivity and specificity [18]. Linear regression was conducted to explore the effects of body weight on the size of the pituitary gland and brain. A *p* value of <0.05 was considered statistically significant.

## 3 Results

### 3.1 Subjects

A total of 96 dogs with pre‐ and post‐contrast CT scans were initially reviewed for inclusion in the pituitary macrotumor group for this study. A total of 89 cases met the inclusion criteria. Seven cases were excluded because of missing skull data on the CT scan (*n* = 5) or because the CT scan could not be retrieved from the electronic PACS system for review (*n* = 2) due to a recognized period of data loss occurring during an institutional data migration. Of the 89 cases, 58 were brachycephalic, 29 were mesocephalic, and 2 were dolichocephalic. Clinical findings compatible with hypercortisolism, such as polyuria, polyphagia, symmetric alopecia, and supportive laboratory tests, were present in 51 cases. Neurological signs, such as circling, obtundation, visual impairments, and collapse, were present in 52 cases. In 24 cases, dogs had both neurological signs present at the time of imaging and a diagnosis of hypercortisolism. In 10 cases, pituitary macrotumor was an incidental finding on CT imaging. In all cases where an imaging‐diagnosed pituitary tumor was diagnosed, the height of the pituitary gland extended above the height of the sella turcica. The median age for all dogs was 9 years (range 0–14 years), and the median weight was 22.4 kg (range 2.5–54.6 kg). A total of 89 controls matched to cases were included for comparison. Of the controls, 56 were breed matches to the cases. The remaining 33 controls had a skull morphology that matched the cases. The mean age of all the controls was 9.4 years (SD ± 2.5 years), and the median weight was 21.8 kg (range 2.2–67.1 kg).

### 3.2 Measurements

In all cases, the height of the pituitary macrotumor was greater than the height of the sella turcica. The sella turcica in one dog was challenging to measure and contour because it was obscured by a mineralized pituitary mass. The pituitary macrotumors varied widely in shape across all cases, with many being round and spherical with smooth margins, whereas others were irregular with indistinct margins. In all cases, the base of the contrast‐enhancing mass corresponded to the base of the sella turcica. Because most macrotumors were strongly contrast‐enhancing, they were easily distinguished from adjacent structures. However, for cystic macrotumors, margins were not as readily defined.

The results for sella turcica measurements are summarized in Table 1. The sella turcica length (*p* = 0.006) and volume (*p* = 0.006) in brachycephalic dogs were significantly smaller compared to those of non‐brachycephalic dogs.

**TABLE 1:** Sella turcica measurements from computed tomography (CT) images compared across skull morphologies in the 89 control dogs included.

Measurement	Brachycephalic (*N* = 58)	Mesocephalic (*N* = 29)	Dolichocephalic (*N* = 2)	*p* value
Median ST height (cm) and range	0.4 (0.3–0.5)	0.4 (0.3–0.5)	0.45 (0.35–0.55)	0.78
Mean ST width (cm) and SD	0.85 (±0.26)	0.90 (±0.18)	0.93 (±0.26)	0.39
Mean ST length (cm) and SD	0.72 (±0.24)	0.84 (±0.21)	0.75 (±0.3)	0.006
Median ST volume (cm^3^) and range	0.08 (0.05–0.16)	0.13 (0.09–0.23)	0.13 (0.08–0.20)	0.006

*Note*: Data are presented as median (interquartile range) or mean (±standard deviation).

Abbreviation: ST, sella turcica.

Brain measurement data are summarized in Table 2. Significant differences in normal brain measurements were found on the basis of skull morphology for brain volume (*p* = 0.0001), width (*p* = 0.0001), and length (*p* = 0.0001), but not height (*p* = 0.23). The brachycephalic group exhibited the smallest measurements for all significant parameters.

**TABLE 2:** Brain measurements from computed tomography (CT) images compared across skull morphologies in the 89 control dogs included.

Measurement	Combined (*N* = 89)	Brachycephalic (*N* = 58)	Mesocephalic (*N* = 29)	Dolichocephalic (*N* = 2)	*p* value
Median brain height (cm) and range	4.2 (4.0–4.4)	4.2 (3.6–5.3)	4.2 (3.7–4.7)	4.1 (3.9–4.2)	0.23
Median brain width (cm) and range	5.3 (4.1–9.5)	5.1 (4.1–6.1)	5.6 (4.5–9.5)	5.3 (4.5–6.0)	0.0001
Mean brain length (cm) and SD	7.74 (±1.19)	7.26 (±0.98)	8.6 (±1.0)	9.3 (±1.6)	0.0001
Mean brain volume (cm^3^) and SD	82.49 (±19.48)	76.81 (±19.08)	93.1 (±13.4)	92.8 (±46.9)	0.0001

Measurements of the pituitary gland from control dogs are summarized in Table 3. Significant differences in normal pituitary gland dimensions existed between the skull morphologies. The width (*p* = 0.006) and length (*p* = 0.002) of normal pituitary glands were smallest in the brachycephalic group. There was no difference in height between skull morphologies (*p* = 0.28). The mean P:B ratio was 0.22 (SD:0.06), which did not vary across skull morphologies (*p* = 0.09). Normal pituitary gland volume significantly differed across skull morphologies, with the brachycephalic group having both the smallest pituitary gland volume (*p* = 0.0001) and pituitary gland volume to brain volume ratio (*p* = 0.0004).

**TABLE 3:** Normal pituitary gland measurements from computed tomography (CT) images compared across skull morphologies from 89 control dogs.

Measurement	Brachycephalic (*N* = 58)	Mesocephalic (*N* = 29)	Dolichocephalic (*N* = 2)	*p* value
Median PG height (cm) and range	0.4 (0.2–0.6)	0.4 (0.3–0.5)	0.4 (0.3–0.5)	0.20
Median PG width (cm) and range	0.5 (0.4–0.7)	0.7 (0.6–0.8)	0.7 (0.6–0.8)	0.006
Median PG length (cm) and range	0.6 (0.4–0.7)	0.8 (0.6–0.8)	0.65 (0.5–0.8)	0.002
Median PG volume (cm^3^) and range	0.06 (0.03–0.09)	0.11 (0.08–0.15)	0.12 (0.04–0.19)	0.0001
Median PGV:BV ratio and range	0.0007 (0.0005–0.0010)	0.0012 (0.0009–0.0014)	0.0011 (0.0007–0.0015)	0.0004
Mean P:B ratio (standard deviation)	0.21 (0.06)	0.24 (0.06)	0.24 (0.05)	0.09

*Note*: Data are presented as median (interquartile range).

Abbreviations: P:B Ratio, pituitary height to brain area ratio; PG, pituitary gland; PGV:BV Ratio, pituitary gland volume to brain volume ratio.

Data from dogs with a pituitary macrotumor are summarized in Table 4. There were no significant differences in pituitary macrotumor size between the skull morphologies. A total of 21.3% (19/89) of pituitary macrotumors were smaller than 1 cm in diameter.

**TABLE 4:** Pituitary macrotumor measurements from computed tomography (CT) images compared across skull morphologies from the 89 dogs included.

Measurement	Brachycephalic (*N* = 58)	Mesocephalic (*N* = 29)	Dolichocephalic (*N* = 2)	*p* value
Mean PG height (cm) and SD	1.48 (±0.50)	1.36 (±0.55)	1.05 (±0.21)	0.33
Mean PG width (cm) and SD	1.36 (±0.42)	1.32 (±0.43)	1.00 (±0.14)	0.49
Mean PG length (cm) and SD	1.51 (±0.53)	1.42 (±0.48)	0.95 (±0.7)	0.30
Median PG volume (cm^3^) and range	1.73 (0.62–2.73)	1.50 (0.50–2.86)	0.55 (0.54–0.56)	0.48
Mean PGV:BV ratio and SD	0.021 (0.0088–0.037)	0.017 (0.0049–0.029)	0.0078 (0.0053–0.029)	0.39
Mean P:B ratio (standard deviation)	0.83 (0.27)	0.78 (0.31)	0.67 (0.23)	0.59

*Note*: Data are presented as median (interquartile range) or mean (± standard deviation).

Abbreviations: P:B Ratio, pituitary height to brain area ratio; PG, pituitary gland; PGV:BV ratio, pituitary gland volume to brain volume ratio.

**FIGURE 4:**
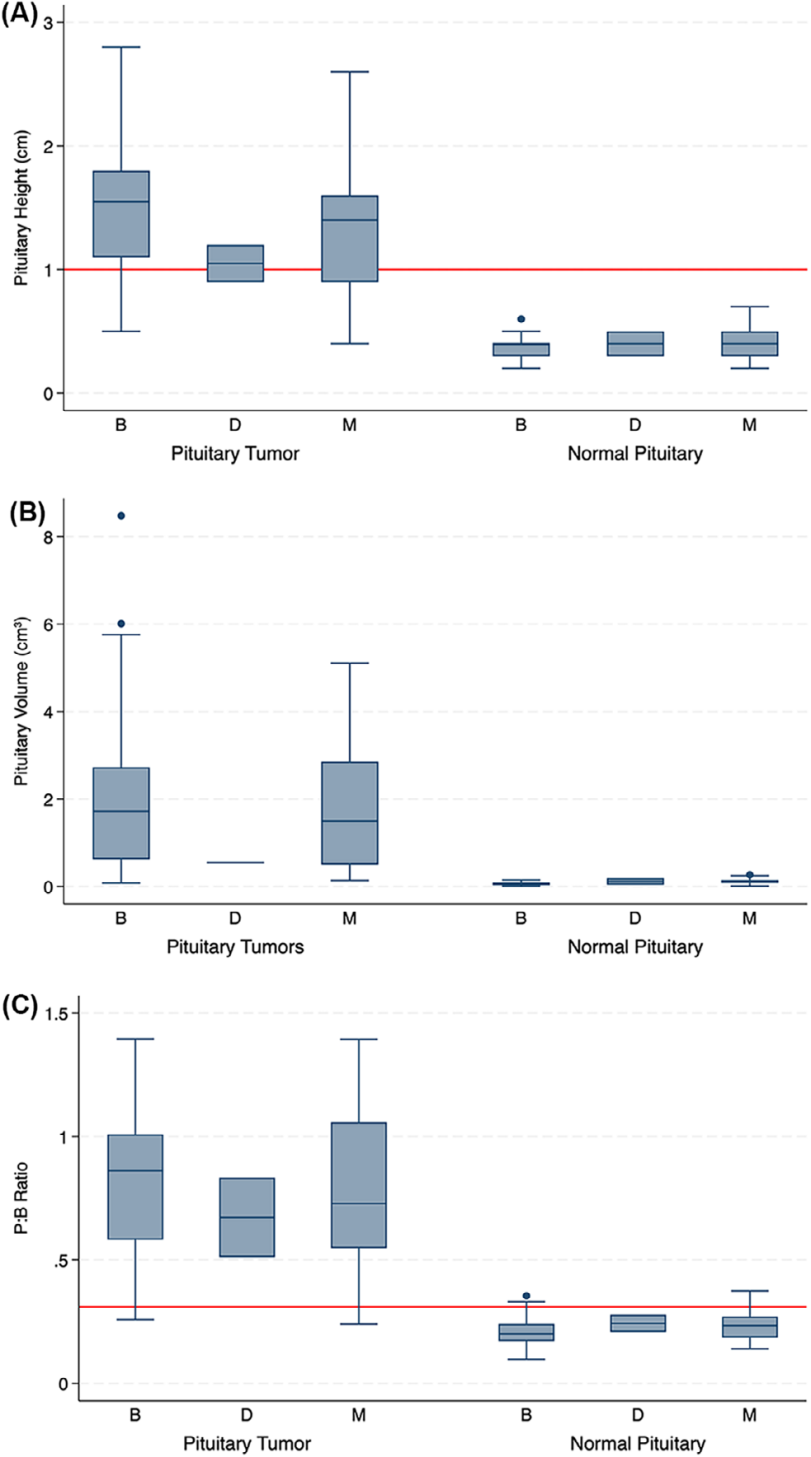
Box and Whisker plots of pituitary macrotumors (*n* = 89) and normal pituitary glands (*n* = 89) across different skull morphologies. (A) Box plot showing pituitary height (cm). (B) Box plot showing pituitary volume (cm^3^). (C) Box plot showing P:B ratio. The red line represents a height of 1 cm in part (A) and the cutoff value of 0.31 for the P:B ratio in part (C). B, brachycephalic; D, dolichocephalic; M, mesocephalic.

Comparisons of pituitary heights and volumes across skull morphologies are shown in Figure 4. Following ROC curve and Liu method analysis, a pituitary gland height of >0.60 cm or a pituitary volume of >0.17 cm^3^ should be considered a macrotumor in brachycephalic dogs. For mesocephalic dogs, a pituitary gland height of >0.65 cm or a pituitary volume of >0.31 cm^3^ should be regarded as a macrotumor. Threshold values for dolichocephalic dogs could not be calculated due to an insufficient sample size.

### 3.3 Body Weight and Pituitary Size

**FIGURE 5:**
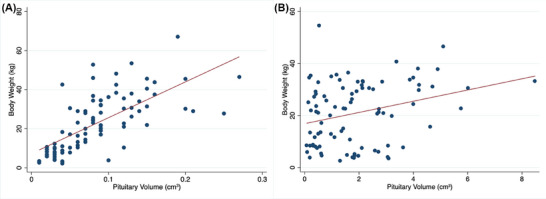
Relationship between pituitary volume (cm^3^) and body weight (kg) in dogs. (A) Scatter plot showing the relationship between body weight (kg) and normal pituitary gland volume (kg) along with the fitted regression line. The results show a significant positive association (Coef.: 182.80, 95% CI 140.48–225.13, *p* < 0.0001). (B) Scatter plot showing the relationship between body weight (kg) and pituitary macrotumor volume (cm^3^) along with its fitted regression line. The results show a significant positive association (Coef.: 3.95, 95% CI 0.61–7.30, *p* < 0.0001).

Because brain size scales with weight, but pituitary size has been relatively conserved in dogs, a weight‐aware pituitary and brain morphometry assessment may reduce misclassification at weight and size extremes in dogs, allow for standardized imaging thresholds across centers, and improve future prognostic models [5, 12, 18, 19]. To explore the effect of body weight on pituitary gland and brain size, linear regression was performed. For dogs with normal pituitary glands, the overall model was significant (adjusted *R* ^2^ = 0.46, *p* < 0.0001). Pituitary volume was positively associated with increasing body weight (Coef.: 182.80, 95% CI 140.48–225.13, *p* < 0.0001; Figure 5A). However, the pituitary height (*p* = 0.18), width (*p* = 0.45), and length (*p* = 0.65) did not significantly alter the model. Similarly, for dogs with pituitary macrotumors, the regression model was significant (adjusted *R* ^2^ = 0.11, *p* = 0.009), with pituitary volume significantly positively associated with body weight (Coef.: 3.95, 95% CI 0.61–7.30, *p* < 0.0001; Figure 5B). Pituitary height (*p* = 0.21), width (*p* = 0.16), and length (*p* = 0.18) did not have significant effects on the model.

**FIGURE 6:**
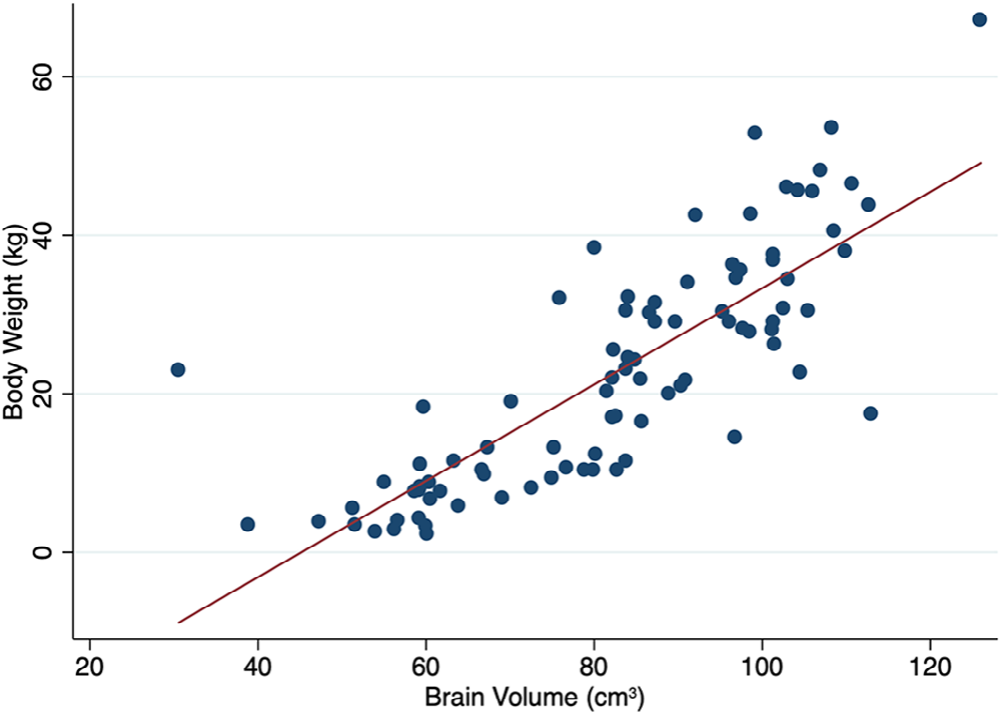
Relationship between brain volume (cm^3^) and body weight in dogs with normal pituitary glands. Scatter plot with the fitted line from the linear regression showing the relationship between body weight and brain volume. The results show a significant positive association (adjusted *R* ^2^ = 0.73, Coef.: 0.30 95% CI 0.15–0.46, *p* < 0.0001).

In all dogs, those with pituitary macrotumors and normal pituitary glands combined, the overall model was also significant (*p* < 0.0001, *R* ^2^ = 0.77). Body weight had a significant positive association with brain volume (*p* = 0.001; Figure 6), brain length (*p* < 0.0001), and brain width (*p* < 0.0001). However, brain height did not affect the model (*p* = 0.64).

Body weight effects on brain size in dogs with normal pituitary glands were also evaluated. The overall model was significant (*p* < 0.0001, adjusted *R* ^2^ = 0.73). Body weight was positively associated with brain volume (Coef.: 0.30, 95% CI 0.15–0.46, *p* < 0.0001), brain length (Coef.: 4.63, 95% CI 2.52–6.73, *p* < 0.0001), and brain width (Coef.: 4.08, 95% CI 1.15–7.00, *p* = 0.007). Brain height did not affect the model (*p* = 0.57). For brachycephalic dogs, the overall model was significant (*p* < 0.0001, adjusted *R* ^2^ = 0.74). Increasing body weight had a significant positive association with increasing brain volume (Coef.: 0.28, 95% CI 0.12–0.44, *p* = 0.001) and brain length (Coef.: 8.07, 95% CI 5.36–10.78, *p* < 0.0001). Brain width (*p* = 0.10) and height (*p* = 0.15) did not significantly affect the model. For mesocephalic dogs, the overall model was significant (*p* < 0.0001, adjusted *R* ^2^ = 0.67). Increasing body weight had a significant positive association with increasing brain volume (Coef.: 0.65, 95% CI 0.21–1.09, *p* = 0.006). Brain length (*p* = 0.99), brain width (*p* = 0.05), and brain height (*p* = 0.63) did not have a significant effect on the model. Linear regression was not performed for dolichocephalic dogs due to an insufficient sample size.

Body weight effects on brain size in dogs with pituitary macrotumors were also looked at. The overall model was significant (adjusted *R* ^2^ = 0.77, *p* < 0.0001). Body weight had a significant positive association with brain length (Coef.: 7.24, 95% CI 5.54–8.94, *p* < 0.0001) and brain width (Coef.: 5.80, 95% CI 3.63–7.97, *p* < 0.0001). Brain volume (*p* = 0.83) and height (*p* = 0.29) did not significantly affect the model.

To evaluate the effect of body weight on brain size across unique skull morphologies, only the control group was analyzed to avoid intrinsic error in the case group, where pituitary masses may have caused a compression of the adjacent brain. For brachycephalic dogs, the overall model was significant (adjusted *R* ^2^ = 0.74, *p* < 0.0001). Increasing body weight was positively associated with an increased brain volume (Coef.: 0.28, 95% CI 0.12–0.44) and brain length (Coef.: 8.07, 95% CI 5.36–10.78, *p* < 0.0001). Brain height (*p* = 0.15) and width (*p* = 0.10) did not significantly affect the model. For mesocephalic dogs, the overall model evaluating the effects of body weight on measured brain parameters was significant (adjusted *R* ^2^ = 0.67, *p* < 0.0001). Increasing body weight was positively associated with increasing brain volume (Coef.: 0.65, 95% CI 0.21–1.09, *p* = 0.006). Brain length (*p* = 0.99), height (*p* = 0.63), and width (*p* = 0.05) did not significantly alter the model. Linear regression was not performed for dolichocephalic dogs due to an insufficient sample size.

## 4 Discussion

A precise definition of canine pituitary macrotumors based on CT imaging is essential because CT provides the mainstay for diagnosis, monitoring and treatment regimens, response assessment, and identification of prognostic factors. Treatment options include radiation therapy and transsphenoidal hypophysectomy, thus requiring accurate tumor size determination. Because a definitive histopathologic diagnosis is not widely or routinely feasible, tracking biologic behavior using CT imaging over time is vital to identify dogs that may benefit from novel therapeutic strategies.

This study assessed 178 contrast CT images from normal dogs and dogs radiologically diagnosed with pituitary macrotumors to define the size variation in the sella turcica, normal pituitary glands, and pituitary macrotumors across different skull morphologies. The sella turcica was significantly smaller in length and volume in brachycephalic dogs compared to mesocephalic and dolichocephalic dogs. Although no significant difference in normal pituitary gland height was identified across skull morphologies, brachycephalic dogs had significantly smaller width, length, and volume than non‐brachycephalic dogs. The pituitary volume to brain volume ratio was also significantly smaller in brachycephalic dogs. Despite that brachycephalic pituitary gland width, length, and volume were significantly smaller than non‐brachycephalic dogs, the volume of pituitary macrotumors was not significantly different across skull morphologies. This may in part be due to different growth characteristics or the timing of diagnosis (diagnostic bias). A total of 19 (21%) of the 89 dogs with pituitary macrotumors were smaller than 1 cm in diameter, supporting the need to adopt unique criteria suitable for diverse dog populations as opposed to utilizing human guidelines. On the basis of these findings, we propose new criteria for the radiological diagnosis of canine pituitary macrotumors. In brachycephalic dogs, a pituitary gland could be considered a macrotumor when its height exceeds 0.60 cm or its volume exceeds 0.17 cm^3^. In mesocephalic dogs, a pituitary gland could be regarded as a macrotumor when its height exceeds 0.65 cm or its volume exceeds 0.31 cm^3^. Further studies should seek to validate these proposed cutoffs, to define radiological parameters for dolichocephalic dogs, and to clarify if there is a clinical impact on monitoring or therapeutic decision‐making. These proposed values are not intended to equate to clinical status or portend a negative outcome. Rather, these efforts to establish smaller cutoffs may have the potential to aid in monitoring and therapeutic stratification in future studies but are not intended to alter existing therapeutic strategies. Some dogs may be monitored for pituitary‐dependent hypercortisolism without an identifiable pituitary mass on CT or MRI for clinicians when using 1 cm as a cutoff. Yet, these dogs have the potential to develop progressive endocrine disease and/or neurologic signs and may benefit from more frequent monitoring [20]. Establishing a smaller, more representative cutoff may enable more dogs to be referred earlier for surgery or radiation therapy, prior to the development of neurological signs. Importantly, dogs with smaller tumors may carry a better prognosis following definitive surgery or radiation therapy compared to dogs with larger tumors, supporting that diagnosis earlier may impact outcome [12, 21, 22, 23, 24]. Additionally, 3D volumetric analysis may better aid in predicting risks unique to skull type for surgery or radiation therapy, as they better capture extension to adjacent structures [25]. Given that survival in dogs with untreated pituitary tumors can range from 48 to 916 days, establishing smaller cutoffs will also allow monitoring studies to better capture potential variables that impact tumor growth characteristics [23]. Finally, the adoption of a more representative cutoff that favors morphology‐aware thresholds may be useful in clinical research that seeks to use control cohorts or stratified randomization efforts to evaluate treatment effects.

The measurements of normal pituitary gland dimensions in this study were similar to those in previous studies. In this study, the median pituitary gland height was 0.42 cm (IQR: 0.37), the median width was 0.6 cm (IQR: 0.2), and the median length was 0.6 cm (IQR: 0.3). Studies from 2004 and 2006 reported similar mean pituitary gland heights, widths, and lengths [6, 11]. Other studies also had similar results with slight differences. In a 2001 study, the mean height was slightly larger, and the mean width was slightly smaller than our findings [5]. In a 2018 study, the mean width and height were slightly smaller, and the mean length was slightly larger than our findings [6]. These minor differences may be attributed to differences in sample population, image acquisition and processing, or interobserver variation. We also assessed the P:B ratio in this study. The mean P:B ratio of the normal pituitary gland was 0.22 (SD: 0.06), which is close to the previously reported 0.21 (SD: 0.03) [6]. The P:B ratio did not vary across skull morphology, although this may represent a Type 2 error, and with a larger data set, this measurement might prove useful in developing skull type‐specific measurements for the normal pituitary.

Some limitations in our study support further evaluation of pituitary size. Prospective studies are ideal to avoid potential inaccuracies during data recording, such as incomplete clinical histories, or standardize the imaging diagnosis of pituitary macrotumor. A prospective design may also allow thin slice MRI imaging, which is generally better at detecting subtle soft tissue changes compared to CT. Because pituitary macrotumor volumes are larger on post‐contrast MRI compared to CT [26], measurements reported may have underestimated the actual size. Case selection in our study was based on an imaging‐diagnosis of pituitary macrotumors because tissue diagnosis was not possible to entirely rule out other tumors, such as meningioma, lymphoma, craniopharyngioma, and granular cell tumor, that could affect the sella turcica [27, 28]. This reflects current veterinary practice, in which antemortem pituitary tumor biopsy is infrequently performed, and imaging is depended upon for clinical decision‐making. To mitigate misclassification, we employed a multi‐criteria definition, which included the presence of a sellar mass with suprasellar/parasellar extension, typical contrast enhancement, and clinical/endocrine corroboration. We also ensured that all CT datasets were reviewed by a board‐certified radiologist. Future studies that include imaging‐diagnosed cases without histopathologic confirmation could use clinical response and outcome data to support a diagnosis of macrotumor; this was not feasible in this dataset due to variations in treatment and lack of consistent follow‐up. Finally, there were few dolichocephalic dogs to evaluate, and not all cases could be breed‐matched in the control group.

These data provide a robust foundation for classifying canine pituitary macrotumors on CT imaging based on the skull conformation. The establishment of new thresholds by which to define macrotumor should be validated in future work, ultimately aiding in the detection, treatment, and response assessment of dogs with pituitary neoplasms.

## Author Contributions

Conception and design: Hannah V. Pham, Michael S. Kent, and Kelsey D. Brust. Acquisition of data: Hannah V. Pham, Michael S. Kent, and Kelsey D. Brust. Analysis and interpretation of data: Hannah V. Pham, Michael S. Kent, Kelsey D. Brust, Matthias Rosseel, and Jessica A. Lawrence. Drafting the article: Hannah V. Pham, Michael S. Kent, Kelsey D. Brust, Matthias Rosseel, and Jessica A. Lawrence. Revising article for intellectual content: Hannah V. Pham, Michael S. Kent, Kelsey D. Brust, Matthias Rosseel, and Jessica A. Lawrence. Final approval of the completed article: Hannah V. Pham, Michael S. Kent, Kelsey D. Brust, Matthias Rosseel, and Jessica A. Lawrence.

## Disclosure

Abstract presented at the 2024 National Veterinary Scholars Symposium in Saint Paul, MN, USA and the 2024 Students Training in Advanced Research Poster Presentation in Davis, CA, USA. A reporting checklist was not used due to the retrospective nature of this article.

## Conflicts of Interest

The authors declare no conflicts of interest.

## Data Availability

The data that support the findings of this article are available upon reasonable request from the corresponding author, M.S.K.

## References

[1] K. Sanders , S. Galac , and B. P. Meij , “Pituitary Tumour Types in Dogs and Cats,” Veterinary Journal 270 (2021): 105623, 10.1016/j.tvjl.2021.105623. 33641809

[2] M. E. Molitch and E. J. Russell , “The Pituitary “Incidentaloma”,” Annals of Internal Medicine 112, no. 12 (1990): 925–931, 10.7326/0003-4819-112-12-925. 2187392

[3] S. A. Moore and D. P. O'Brien , “Canine Pituitary Macrotumors,” Compendium: Continuing Education for Veterinarians 30, no. 1 (2008): 33–40; quiz 41. 18278746

[4] M. Araujo‐Castro , A. A. Cancela , C. Vior , E. Pascual‐Corrales , and V. Rodriguez Berrocal , “Radiological Knosp, Revised‐Knosp, and Hardy‐Wilson Classifications for the Prediction of Surgical Outcomes in the Endoscopic Endonasal Surgery of Pituitary Adenomas: Study of 228 Cases,” Frontiers in Oncology 11 (2021): 807040, 10.3389/fonc.2021.807040. 35127519 PMC8810816

[5] H. Kippenes , P. R. Gavin , S. L. Kraft , R. D. Sande , and R. L. Tucker , “Mensuration of the Normal Pituitary Gland From Magnetic Resonance Images in 96 Dogs,” Veterinary Radiology & Ultrasound: The Official Journal of the American College of Veterinary Radiology and the International Veterinary Radiology Association 42, no. 2 (2001): 130–133, 10.1111/j.1740-8261.2001.tb00915.x. 11327360

[6] S. Nadimi , M. Molazem , and S. Jarolmasjed , “Volumetric Evaluation of Pituitary Gland in Dog and Cat Using Computed Tomography,” Veterinary Research Forum 9, no. 4 (2018): 337–341, 10.30466/vrf.2018.33073. 30713612 PMC6346484

[7] M. Suzuki , T. Takashima , M. Kadoya , et al., “Height of Normal Pituitary Gland on MR Imaging: Age and Sex Differentiation,” Journal of Computer Assisted Tomography 14, no. 1 (1990): 36–39, 10.1097/00004728-199001000-00006. 2298994

[8] H. S. Kooistra , G. Voorhout , J. A. Mol , and A. Rijnberk , “Correlation Between Impairment of Glucocorticoid Feedback and the Size of the Pituitary Gland in Dogs With Pituitary‐Dependent Hyperadrenocorticism,” Journal of Endocrinology 152, no. 3 (1997): 387–394, 10.1677/joe.0.1520387. 9071959

[9] M. Menchetti , L. De Risio , G. Galli , et al., “Neurological Abnormalities in 97 Dogs With Detectable Pituitary Masses,” Veterinary Quarterly 39, no. 1 (2019): 57–64, 10.1080/01652176.2019.1622819. 31112462 PMC6831018

[10] R. H. van der Vlugt‐Meijer , B. P. Meij , and G. Voorhout , “Dynamic Computed Tomographic Evaluation of the Pituitary Gland in Healthy Dogs,” American Journal of Veterinary Research 65, no. 11 (2004): 1518–1524, 10.2460/ajvr.2004.65.1518. 15566090

[11] R. H. van der Vlugt‐Meijer , B. P. Meij , and G. Voorhout , “Intraobserver and Interobserver Agreement, Reproducibility, and Accuracy of Computed Tomographic Measurements of Pituitary Gland Dimensions in Healthy Dogs,” American Journal of Veterinary Research 67, no. 10 (2006): 1750–1755, 10.2460/ajvr.67.10.1750. 17014327

[12] S. J. van Rijn , S. Galac , M. A. Tryfonidou , et al., “The Influence of Pituitary Size on Outcome after Transsphenoidal Hypophysectomy in a Large Cohort of Dogs With Pituitary‐Dependent Hypercortisolism,” Journal of Veterinary Internal Medicine 30, no. 4 (2016): 989–995, 10.1111/jvim.14367. 27425149 PMC5108476

[13] G. Galli , G. Bertolini , G. Dalla Serra , and M. Menchetti , “Suspected Pituitary Apoplexy: Clinical Presentation, Diagnostic Imaging Findings and Outcome in 19 Dogs,” Veterinary Sciences 9, no. 4 (2022): 191, 10.3390/vetsci9040191. 35448689 PMC9026492

[14] W. Sokolowski , K. Barszcz , M. Kupczynska , et al., “Morphometry and Morphology of Rostral Cranial Fossa in Brachycephalic Dogs—CT Studies,” PLoS ONE 15, no. 10 (2020): e0240091, 10.1371/journal.pone.0240091. 33002083 PMC7529308

[15] K. L. van Bokhorst , H. S. Kooistra , S. Boroffka , and S. Galac , “Concurrent Pituitary and Adrenocortical Lesions on Computed Tomography Imaging in Dogs With Spontaneous Hypercortisolism,” Journal of Veterinary Internal Medicine 33, no. 1 (2019): 72–78, 10.1111/jvim.15378. 30536676 PMC6335443

[16] C. H. Vite and E. Head , “Aging in the Canine and Feline Brain,” Veterinary Clinics of North America: Small Animal Practice 44, no. 6 (2014): 1113–1129, 10.1016/j.cvsm.2014.07.008. 25441628 PMC4254595

[17] K. Czeibert , A. Sommese , O. Petnehazy , T. Csorgo , and E. Kubinyi , “Digital Endocasting in Comparative Canine Brain Morphology,” Frontiers in Veterinary Science 7 (2020): 565315, 10.3389/fvets.2020.565315. 33134351 PMC7572857

[18] P. Clayton , CUTPT: Stata Module for Empirical Estimation of Cutpoint for a Diagnostic Test (Statistical Software Components, 2013).

[19] M. J. Schmidt , K. H. Amort , K. Failing , M. Klingler , M. Kramer , and N. Ondreka , “Comparison of the Endocranial‐ and Brain Volumes in Brachycephalic Dogs, Mesaticephalic Dogs and Cavalier King Charles spaniels in Relation to Their Body Weight,” Acta Veterinaria Scandinavica 56, no. 1 (2014): 30, 10.1186/1751-0147-56-30. 24886598 PMC4038113

[20] E. H. Bertoy , E. C. Feldman , R. W. Nelson , A. B. Dublin , M. H. Reid , and M. S. Feldman , “One‐Year Follow‐Up Evaluation of Magnetic Resonance Imaging of the Brain in Dogs With Pituitary‐Dependent Hyperadrenocorticism,” Journal of the American Veterinary Medical Association 208, no. 8 (1996): 1268–1273. 8635969

[21] J. M. Hanson , E. Teske , G. Voorhout , S. Galac , H. S. Kooistra , and B. P. Meij , “Prognostic Factors for Outcome After Transsphenoidal Hypophysectomy in Dogs With Pituitary‐Dependent Hyperadrenocorticism,” Journal of Neurosurgery 107, no. 4 (2007): 830–840, 10.3171/JNS-07/10/0830. 17937231

[22] J. M. Hanson , H. M. van 't , G. Voorhout , E. Teske , H. S. Kooistra , and B. P. Meij , “Efficacy of Transsphenoidal Hypophysectomy in Treatment of Dogs With Pituitary‐dependent Hyperadrenocorticism,” Journal of Veterinary Internal Medicine 19, no. 5 (2005): 687–694, 10.1892/0891-6640(2005)19[687:eothit]2.0.co;2. 16231713

[23] M. S. Kent , D. Bommarito , E. Feldman , and A. P. Theon , “Survival, Neurologic Response, and Prognostic Factors in Dogs With Pituitary Masses Treated With Radiation Therapy and Untreated Dogs,” Journal of Veterinary Internal Medicine 21, no. 5 (2007): 1027–1033, 10.1892/0891-6640(2007)21[1027:snrapf]2.0.co;2. 17939560

[24] A. P. Theon and E. C. Feldman , “Megavoltage Irradiation of Pituitary Macrotumors in Dogs With Neurologic Signs,” Journal of the American Veterinary Medical Association 213, no. 2 (1998): 225–231. 9676592

[25] L. L. Van Stee , S. J. Van Rijn , S. Galac , and B. P. Meij , “Challenges of Transsphenoidal Pituitary Surgery in Severe Brachycephalic Dogs,” Frontiers in Veterinary Science 10 (2023): 1154617, 10.3389/fvets.2023.1154617. 37408830 PMC10318542

[26] T. L. Gieger and M. W. Nolan , “Treatment Outcomes and Target Delineation Utilizing CT and MRI in 13 Dogs Treated With a Uniform Stereotactic Radiation Therapy Protocol (16 Gy single fraction) for Pituitary Masses: (2014–2017),” Veterinary and Comparative Oncology 19, no. 1 (2021): 17–24, 10.1111/vco.12627. 32548944

[27] M. A. Miller , D. S. Bruyette , J. C. Scott‐Moncrieff , et al., “Histopathologic Findings in Canine Pituitary Glands,” Veterinary Pathology 55, no. 6 (2018): 871–879, 10.1177/0300985818766211. 29665752

[28] L. Polledo , G. C. M. Grinwis , P. Graham , M. Dunning , and K. Baiker , “Pathological Findings in the Pituitary Glands of Dogs and Cats,” Veterinary Pathology 55, no. 6 (2018): 880–888, 10.1177/0300985818784162. 29929454

